# Species-specific reservoir effect estimates: A case study of archaeological marine samples from the Bering Strait

**DOI:** 10.1177/09596836211041728

**Published:** 2021-09-06

**Authors:** Jack PR Dury, Gunilla Eriksson, Arkady Savinetsky, Maria Dobrovolskaya, Kirill Dneprovsky, Alison JT Harris, Johannes van der Plicht, Peter Jordan, Kerstin Lidén

**Affiliations:** 1 Department of Archaeology and Classical Studies, Stockholm University, Sweden; 2 Arctic Centre, University of Groningen, The Netherlands; 3 Severtsov Institute of Ecology and Evolution, Russian Academy of Sciences, Russia; 4 Institute of Archaeology, Russia; 5 State Museum for Oriental Art, Russia; 6 BioArCh, Department of Archaeology, University of York, UK; 7 Faculty of Science and Engineering, University of Groningen, The Netherlands; 8 Department of Archaeology and Ancient History, Lund University, Sweden; 9 Global Station for Indigenous Studies and Cultural Diversity (GSI), GI-CoRE, Hokkaido University, Japan

**Keywords:** Bering Strait, Ekven, marine reservoir effects, Old Bering Sea Culture, radiocarbon, reservoir age, Δ*R*

## Abstract

Due to the marine reservoir effect, radiocarbon dates of marine samples require a correction. Marine reservoir effects, however, may vary among different marine species within a given body of water. Factors such as diet, feeding depth and migratory behaviour all affect the ^14^C date of a marine organism. Moreover, there is often significant variation within single marine species. Whilst the careful consideration of the Δ*R* values of a single marine species in a given location is important, so too is the full range of Δ*R* values within an ecosystem. This paper illustrates this point, using a sample pairing method to estimate the reservoir effects in 17 marine samples, of eight different species, from the archaeological site of Ekven (Eastern Chukotka, Siberia). An OxCal model is used to assess the strength of these estimates. The marine reservoir effects of samples passing the model range from Δ*R* (Marine20) = 136 ± 41–Δ*R* = 460 ± 40. Marine reservoir effect estimates of these samples and other published samples are used to explore variability in the wider Bering Strait region. The archaeological implications of this variability are also discussed. The calibrating of ^14^C dates from human bone collagen, for example, could be improved by applying a dietary relevant marine reservoir effect correction. For humans from the site of Ekven, a Δ*R* (Marine20) correction of 289 ± 124 years or reservoir age correction of 842 ± 123 years is suggested.

## Introduction

### Marine reservoir effects

Terrestrial organisms acquire carbon from the atmosphere (either directly or indirectly); however, there are other ‘reservoirs’ of radiocarbon, such as marine bodies of water. The ^14^C/^12^C ratios of these reservoirs can differ from the ^14^C/^12^C ratio in the atmosphere. Aquatic organisms generally have a lower ^14^C/^12^C ratio than atmospheric CO_2_, leading to samples yielding older ‘apparent ages’. For marine organisms, this ‘marine reservoir effect’ (MRE) can be many 100s of years (Heaton et al., 2020). For freshwater environments, the reservoir effect can vary widely, being very large (Philippsen, 2013) or not present at all (Dury et al., 2018; van der Plicht et al., 2020), depending on geochemical circumstances (Mook, 2005).

The MRE exists due to the long residence times of ^14^C in deep ocean waters. Whereas surface-water carbon is in exchange with atmospheric carbon, this is not the case for deep waters. Due to deep water upwelling in certain locations, surface ocean waters also become depleted in ^14^C (Alves et al., 2018). Globally, therefore, MREs are not uniform. To accurately date marine samples, ^14^C dates must be calibrated either against a reservoir-age adjusted terrestrial calibration curve (Reimer et al., 2020) or a Δ*R* adjusted marine calibration curve (Heaton et al., 2020). The reservoir age, *R*(*t*), of such a sample, is defined as the difference between the measured ^14^C age of an aquatic sample and that of a contemporaneous terrestrial (atmospheric) sample (Stuiver et al., 1986). Δ*R* is the difference between the ^14^C date calculated by reverse-calibrating the calendar date of the terrestrial sample against a marine calibration curve, and the measured ^14^C date of the marine sample (Russell et al., 2011a). Positive or negative Δ*R* values represent an MRE larger or smaller than the global average, respectively. Variability of global MREs is due to many factors. The magnitude of MREs can vary between different marine species (Russell et al., 2011a) due to differences in mobility, diet and feeding depth. MREs can also vary geographically due to factors such as deepwater upwelling and sea ice cover (Heaton et al., 2020). Moreover, the reservoir ages of marine samples can vary temporally (Stuiver and Braziunas, 1993; Stuiver et al., 1986). For this reason, when calibrating ^14^C dates for a consumer with dietary inputs of marine carbon, care should be taken to calculate an appropriate *R*(*t*) or Δ*R* value. These should be local, temporally relevant and take into account the types of species the human consumed. To calibrate the ^14^C date of an individual from a seal-hunting society, for example, the reservoir ages or Δ*R* values of local seals will be more applicable than those of local shellfish. For humans with mixed diets, weighted values should be used to calibrate their ^14^C dates.

### Bering Strait marine reservoir effects

Although the measurement of accurate reservoir effects is important in all contexts, it is particularly pertinent to arctic contexts where MREs can be quite high compared to the global average (Austin et al., 1995). Moreover, in arctic environments, archaeological evidence demonstrates the economic importance of marine fauna for use as food and raw materials. This being the case, groups utilising marine species can be subjected to a range of different MREs. Here, marine samples from the archaeological site of Ekven, located at the Bering Strait, have been investigated to demonstrate the range of MREs within a given geographic location. These data can then be used to create more nuanced chronological reconstructions of human settlement of the Bering and Chukchi Sea coastlines. Ekven belongs to the Old Bering Sea Culture (OBS), ancestral to many subsequent Siberian and Alaskan groups (Flegontov et al., 2019; Mason, 2016). OBS sites are found along the coast of the Chukchi Peninsula, St. Lawrence Island and scattered finds also occur in Alaska (Mason and Rasic, 2019). The OBS archaeological culture developed a sophisticated marine mammal hunting toolkit and may have targeted walrus, bowhead whale, ringed seal and bearded seal (Gusev et al., 1999; Hill, 2011; Whitridge, 1999). Radiocarbon dating of OBS cultural material will, therefore, require detailed knowledge of the local MRE.

A significant number of radiocarbon dates exist for the OBS archaeological culture, pointing towards a date range of c. cal AD 300–1400 (Mason and Rasic, 2019). This dating has been conducted on material from several archaeological sites in the Bering Strait to understand the temporal relationship between different archaeological cultures. To avoid uncertainties associated with the MRE of marine samples, many dates were obtained from charcoal and wood samples instead (Gerlach and Mason, 1992; Gusev et al., 1999). These samples, however, may be subject to the so-called old-wood problem, a dating discrepancy between the date of formation of tree rings and the use of the wood (the dating event) (Schiffer, 1986). This is particularly problematic in arctic contexts where wood is more scarce, driftwood is utilised and wood is readily reused. The dated wood/charcoal samples are not necessarily contemporaneous with the archaeological phase of interest (Cook and Comstock, 2014). Similarly, the direct dating of artefacts may not be helpful; artefacts are often made of marine faunal materials and are therefore subject to the MRE. Moreover, the long use-life of tools or even the working of old faunal material, such as the use and re-use of whalebone in dwelling and ceremonial structures (McCartney and Savelle, 1993), is likely to yield erroneous interpretation of radiocarbon dates (Pitulko, 2000). To refine OBS culture chronologies, there must be an understanding of the MREs around the Bering Strait.

Recognition of the potential for high MREs around the Bering Strait has been long-standing. Not only do MRE values tend to be high in polar regions (Austin et al., 1995), but the Bering Sea is also a region of significant upwelling. Using shellfish of known collection age (McNeely et al., 2006; Robinson and Thompson, 1981) from the ^14^Chrono database (Reimer and Reimer, 2001, 2017) an average reservoir age of 818 ± 100 years is calculated.

**Figure 1.**
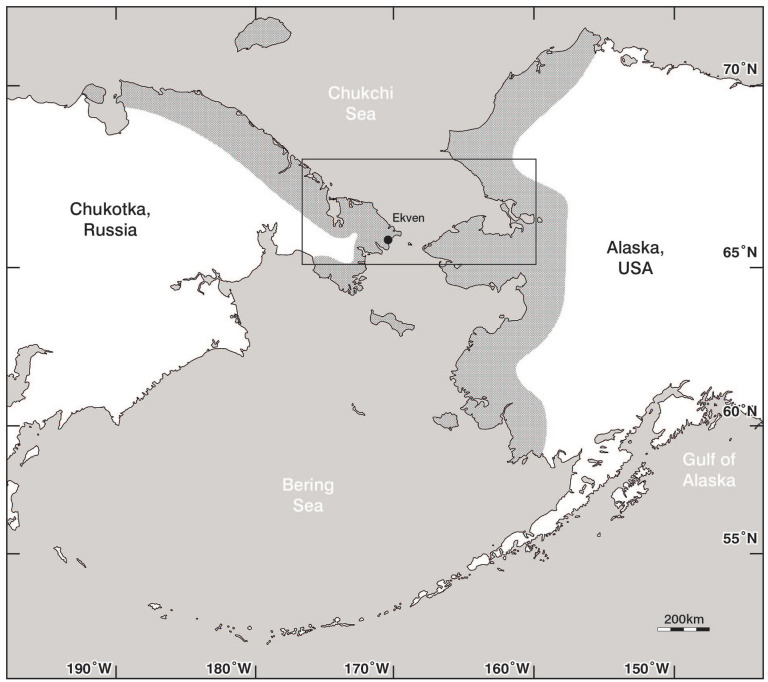
Location of the Ekven mortuary site, the study area of interest (rectangle) and the known distribution of the Old Bering Sea Culture (shaded area, redrawn from Gerlach and Mason, 1992).

These samples, however, do not reflect the entire ecological range of the marine species consumed by humans on the Chukchi coast. Moreover, these samples are not contemporary with the OBS and it remains to be seen if the MREs around the Bering Strait have changed over time. Other investigations have suggested smaller reservoir ages of between 450 and 750 years (Dumond and Griffin, 2002). Several of these MRE measurements, however, were calculated by comparing paired samples of wood or charcoal with marine samples and may have been affected by the old-wood problem. There have been several recent publications researching the MREs of the Bering Strait region, making use of sample pairing protocols. Paired ringed seal and terrestrial sample ^14^C dates from Walakpa, on the northern tip of Alaska (Krus et al., 2019), yield an average *R*(*t*) of 864 ± 105 years. Paired terrestrial and seal sample data from Reuther et al. (2021), from the region in question (Figure 1), yield a reservoir age of 831 ± 132 years.

For the purpose of calibrating radiocarbon dates of human skeletal remains, possible variations in MRE values for different species must be investigated. Abundant archaeological and zooarchaeological evidence from OBS habitation sites, and later ethnographic surveys of Siberian Yupiget, indicate that large marine mammals (walrus and whales), and piscivorous and benthic-feeding seals were the predominant sources of dietary protein and lipids, with smaller contributions from birds, shellfish, terrestrial game and seasonally available plants (Gusev et al., 1999; Hill, 2011; Kozlov et al., 2007; Mason, 2016; Whitridge, 1999; Zdor et al., 2010). The different ecological niches occupied by these taxa are likely to result in different reservoir ages.

## Materials and methods

### Sampling site: Ekven mortuary site

The site of Ekven (Figure 1) is one of the largest OBS culture sites in Chukotka. Over 150 burials have been excavated and these include single and multiple interments, but 60% of the mortuary site and 90% of the habitation site has not been excavated (Commission of the Russian Federation for UNESCO, 2019). Multiple archaeological excavations of the Ekven site (Arutiunov and Sergeev, 1990; Bronshtein, 1991) found OBS burials that often feature a large assemblage of faunal material (both marine and terrestrial species). If we assume that these were contemporary with the burial event, this allows paired dating of marine and terrestrial materials, and these samples can be used to measure species-specific reservoir effects. In total, 13 marine and 25 terrestrial skeletal elements, from 18 closed grave contexts, were sampled for ^14^C dating, as well as δ^13^C and δ^15^N analysis (Table 1). The sampling was limited by the availability of curated material. Of the curated materials, no artefacts or worked faunal elements were selected for analysis. This precluded the possibility that the artefacts were used for many years before their eventual deposition.

**Table 1.** Sampled marine and terrestrial fauna δ^15^N, δ^13^C and ^14^C data.

Sample code	GrM code	Species	Common name	Burial	^14^C Date ± σ (BP)	δ^13^C ± σ (‰)	δ^15^N ± σ (‰)	%C	%N	IRMS C:N
EKV09	15802	Erignathus barbatus	Bearded seal	249	2850 ± 20^1^	−13.4 ± 0.2	+16.0 ± 0.2	44.29	15.35	3.4^2,c^
EKV11	15596	Erignathus barbatus	Bearded seal	250	2190 ± 20^1^	−13.4 ± 0.2	+16.4 ± 0.1	41.28	14.23	3.4^1,a^
EKV12	13655	Pusa hispida	Ringed seal	250	2140 ± 45^2^	−14.0 ± 0.2	+17.8 ± 0.2	23.27	7.63	3.6^2,b^
EKV15	15804	Pusa hispida	Ringed seal	234	2155 ± 35^1^	−14.4 ± 0.2	+17.3 ± 0.2	42.01	14.60	3.4^2,c^
EKV20	15593	Pusa hispida	Ringed seal	260	3355 ± 25^1^	−13.4 ± 0.2	+19.2 ± 0.1	40.17	14.64	3.2^1,a^
EKV27	15812	Pusa hispida	Ringed seal	317	2025 ± 20^2^	−13.0 ± 0.2	+17.2 ± 0.2	44.82	15.58	3.4^2,c^
EKV28	13647	Pusa hispida	Ringed seal	317	2010 ± 25^1^	−14.0 ± 0.2	+16.2 ± 0.2	44.54	15.63	3.3^2,c^
EKV13	15598	Pusa hispida	Ringed seal	250	2140 ± 20^1^	−11.7 ± 0.2	+20.9 ± 0.1	40.20	14.70	3.2^1,a^
EKV01	15797	Odobenus rosmarus	Walrus	279	1580 ± 19^1^	−13.5 ± 0.2	+11.7 ± 0.2	44.78	15.10	3.5^2,c^
EKV14	13653	Odobenus rosmarus	Walrus	250	2050 ± 25^1^	−12.2 ± 0.2	+12.3 ± 0.2	37.66	13.71	3.2^1,b^
EKV29	15814	Odobenus rosmarus	Walrus	317	1985 ± 20^1^	−12.7 ± 0.2	+15.2 ± 0.2	33.61	12.11	3.2^1,b^
EKV26	15810	Uria lomvia	Thick-billed murre	222	1555 ± 20^1^	−14.4 ± 0.2	+18.1 ± 0.2	44.02	15.27	3.4^2,c^
EKV155	21436	*Uria* sp.	Murre	301	2024 ± 24^2^	−14.2 ± 0.2	+18.3 ± 0.3	44.70	15.90	3.3^2,a^
EKV158	21440	*Uria* sp.	Murre	318	2010 ± 24^2^	−14.6 ± 0.2	+19.2 ± 0.3	44.30	15.50	3.3^2,a^
EKV18	15807	Cepphus columba	Pigeon guillemot	214	1875 ± 20^1^	−15.9 ± 0.2	+12.8 ± 0.2	33.94	12.00	3.3^1,b^
EKV157	21438	*Phalacrocorax* sp.	Cormorant	302	1995 ± 24^2^	−12.0 ± 0.2	+18.1 ± 0.3	45.60	16.20	3.3^2,a^
EKV159	21441	*Larus* sp.	Gull	302	1813 ± 24^2^	−15.9 ± 0.2	+17.9 ± 0.3	44.50	15.80	3.3^2,a^
EKV160	21442	*Rissa* sp.	Kittiwake	313	2070 ± 24^2^	−13.6 ± 0.2	+20.0 ± 0.3	45.20	16.00	3.3^2,a^
EKV08	15586	*Anser* sp.	Goose	214	1295 ± 20^1^	−22.3 ± 0.2	+5.8 ± 0.1	41.80	15.21	3.2^1,a^
EKV10	15803	*Anser* sp.	Goose	313	1259 ± 19^1^	−20.6 ± 0.2	+6.7 ± 0.2	34.71	11.88	3.4^1,b^
EKV17	15591	*Anser* sp.	Goose	293	1065 ± 20^1^	−22.7 ± 0.2	+5.9 ± 0.2	44.43	15.34	3.4^2,c^
EKV23	15584	*Anser* sp.	Goose	222	1315 ± 20^1^	−22.2 ± 0.2	+6.4 ± 0.1	41.09	14.92	3.2^1,a^
EKV24	15588	*Anser* sp.	Goose	222	1290 ± 20^1^	−22.3 ± 0.2	+5.3 ± 0.1	39.99	14.42	3.2^1,a^
EKV25	15603	*Anser* sp.	Goose	318	1335 ± 20^1^	−20.3 ± 0.2	+6.9 ± 0.1	39.98	14.58	3.2^1,a^
EKV30	15594	*Anser* sp.	Goose	285/Б/1	859 ± 19^1^	−22.3 ± 0.2	+5.3 ± 0.1	41.46	12.09	4.0^1,a^
EKV32	15817	*Anser* sp.	Goose	318	1300 ± 20^2^	−20.2 ± 0.2	+7.1 ± 0.2	40.14	14.50	3.2^2,b^
EKV33	15601	*Anser* sp.	Goose	301	1220 ± 20^1^	−22.5 ± 0.2	+5.2 ± 0.1	40.04	14.40	3.2^1,a^
EKV34	15585	*Anser* sp.	Goose	325	1205 ± 20^1^	−21.2 ± 0.2	+8.3 ± 0.1	39.61	14.29	3.2^1,a^
EKV35	15818	*Anser* sp.	Goose	279	1345 ± 35^1^	−21.7 ± 0.2	+5.3 ± 0.2	35.42	12.16	3.2^2,b^
EKV36	15819	*Anser* sp.	Goose	283B	1215 ± 25^1^	−21.3 ± 0.2	+7.1 ± 0.2	36.64	12.95	3.3^2,b^
EKV38	15583	*Anser* sp.	Goose	313	1236 ± 19^1^	−21.1 ± 0.2	+6.4 ± 0.1	39.55	14.28	3.2^2,a^
EKV156	21437	*Anser* sp.	Goose	293	1013 ± 24^2^	−22.1 ± 0.2	+6.4 ± 0.3	45.30	16.20	3.3^2,a^
EKV02	13667	Lepus timidus	Mountain hare	317	1186 ± 15^1^	−24.0 ± 0.1	+3.5 ± 0.1	36.98	12.64	3.4^1,a^
EKV04	15799	Lepus timidus	Mountain hare	249	1170 ± 20^1^	−21.3 ± 0.2	+2.7 ± 0.2	44.68	15.43	3.4^1,c^
EKV05	15600	Lepus timidus	Mountain hare	310	1225 ± 20^1^	−21.1 ± 0.2	+2.7 ± 0.1	41.04	14.80	3.2^1,a^
EKV06	15590	Lepus timidus	Mountain hare	310	1252 ± 19^1^	−22.1 ± 0.2	+3.2 ± 0.1	37.13	13.13	3.3^1,a^
EKV07	15800	Lepus timidus	Mountain hare	310	1290 ± 20^1^	−21.5 ± 0.2	+4.0 ± 0.2	41.30	14.47	3.3^1,b^
EKV16	15805	Lepus timidus	Mountain hare	250	3925 ± 25^1^	−21.5 ± 0.2	+1.4 ± 0.2	41.98	14.37	3.4^2,c^
EKV19	15808	Lepus timidus	Mountain hare	234	1258 ± 19^1^	−21.1 ± 0.2	+2.2 ± 0.2	44.71	15.31	3.4^2,c^
EKV39	13657	Lepus timidus	Mountain hare	260	1255 ± 25^1^	−20.8 ± 0.2	+0.9 ± 0.1	37.17	12.95	3.3^2,a^
EKV37	16572	Lagopus lagopus	Willow ptarmigan	302	1175 ± 30^1^	−19.4 ± 0.3	+1.6 ± 0.1	39.02	14.40	3.2^1,a^
EKV03	15798	Rangifer tarandus	Reindeer	282	1394 ± 19^1^	−18.3 ± 0.2	+2.5 ± 0.2	33.33	12.09	3.2^1,b^
EKV22	15595	Rangifer tarandus	Reindeer	250	1219 ± 19^1^	−19.3 ± 0.3	+2.3 ± 0.1	40.16	14.70	3.2^1,a^
EKV31	15815	Rangifer tarandus	Reindeer	317	1241 ± 19^1^	−17.9 ± 0.2	+5.1 ± 0.2	44.64	14.95	3.5^2,c^

Samples in italics fall outside C/N quality control range. Data failing C/N ratio quality control criteria have been struck through. The collagen extraction method for AMS indicated in superscript next to the ^14^C date. The collagen extraction method for IRMS is displayed next to the C/N ratio indicated in superscript followed by the lab of analysis: ^a^Groningen, ^b^Stockholm and ^c^Vilnius.

Additional published data will be considered from within the rectangular area shown in Figure 1. This broad area encompasses the site of Ekven, the area immediately around the Bering Strait and the south of the Chukchi Sea.

### Collagen extraction

Collagen was extracted from bone samples at Stockholm University, applying two variations of the Longin (1971) extraction method. The methods applied to each sample are recorded in Table 1. Method 1: Samples of bone powder were obtained using a dentist’s drill. Surface layers were discarded to avoid contamination. Method 2: Chunks of bone, of roughly 200 mg were removed from the sample using a handheld rotary drill. For both methods, bone collagen was subsequently extracted according to the methods outlined by Brown et al. (1988). This included an ultrafiltration step (Amicon^®^ Ultra-15 Centrifugal Ultracel^®^ Filters) to remove low molecular weight material (<30 kDa). The bone samples were then centrifuged down to 0.5 ml and freeze-dried. Bone collagen was weighed into tin capsules (c. 0.5 mg for carbon and nitrogen isotope ratio analysis, 3.5–5.5 mg for AMS analysis).

### Radiocarbon dating

The dating was performed at the University of Groningen AMS facility. The extracted collagen was combusted to CO_2_ by an elemental analyser, connected to an isotope ratio mass spectrometer (EA/IRMS, Elementar Vario Isotope Cube™/Isoprime 100™). The latter provides the stable isotope ratio ^13^C/^12^C. Part of the CO_2_ was transferred into graphite, by a reaction with H_2_ gas at a temperature of about 600°C, using Fe powder as catalyst (Aerts-Bijma et al., 2001). The graphite was pressed into target holders for the ion source of the AMS. The AMS is a MICADAS-17 (IonPlus^®^) (Mini Carbon Dating System (Synal et al., 2007) manufactured by IonPlus, installed in 2017. The present Groningen laboratory code is GrM.

The ^14^C dates are reported by convention in BP, that is, measured relative to an oxalic acid standard, corrected for isotopic fractionation using the stable isotope ratio ^13^C/^12^C to δ^13^C = −25‰, and using a half-life value of 5568 years (van der Plicht and Hogg, 2006).

The radiocarbon dates were calibrated to calendar ages using OxCal (Bronk Ramsey, 1995), a Bayesian statistical programme utilising the ^14^C date of the measured sample and information from an appropriate ^14^C calibration curve (Bronk Ramsey, 2009a). Depending on the origin of the sample, a terrestrial ‘IntCal20’ curve (Reimer et al., 2020) or marine ‘Marine20’ curve (Heaton et al., 2020) is applied. The Δ*R* values were determined using the Deltar tool (Reimer and Reimer, 2017).

Despite the Bering Strait region being situated below the Arctic Circle, and despite there being a strong current of southerly waters moving north, the use of the Marine20 curve is not recommended at this latitude (Heaton et al., 2020). For the benefit of comparison with other studies, however, for each marine sample, Δ*R* values (using the Marine20 calibration curve) were calculated alongside reservoir ages, *R*(*t*).

### Stable isotope analysis

Stable isotope analysis was conducted using EA/IRMS at three laboratories: University of Groningen Centre for Isotope Research, Stockholm University Department of Geological Sciences and the Vilnius Center for Physical Sciences and Technology. Groningen uses an Elementar Vario Isotope Cube™/Isoprime 100™) EA/IRMS combination. Stockholm uses a Carlo Erba NC2500™ elemental analyser connected to a Finnigan MAT Delta+™ continuous flow IRMS. Vilnius uses an elemental analyser FlashEA 1112™ connected to a ThermoFinnigan Delta Plus advantage™ IRMS.

The isotopic content of materials is expressed in delta (δ) values, which are defined as the deviation (expressed in per mil ‰) of the rare to abundant isotope ratio from that of a reference material:


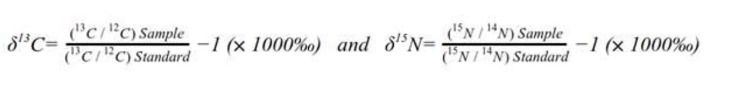


For carbon, the reference material is belemnite carbonate (V-PDB); for nitrogen, the reference is ambient air (Mook, 2005). The error of the stable isotope measurements is typically between 0.05‰ and 0.30‰ for δ^13^C and between 0.10‰ and 0.20‰ for δ^15^N.

The atomic C/N ratio is a proxy for the integrity of the collagen. The widely accepted range of atomic C/N ratios for well-preserved bone and dentine is 2.9–3.6 (DeNiro, 1985), which we have applied here.

## Results

C/N quality control criteria (DeNiro, 1985) was used to assess collagen quality. Carbon and nitrogen concentrations were also considered (Ambrose, 1990; Sealy et al., 2014; van Klinken, 1999) to ensure C/N ratios reflected suitable collagen.

One sample (EKV30) failed to meet published collagen quality control criteria on the basis of C/N ratio (DeNiro, 1985), so δ^13^C and δ^15^N and possibly ^14^C data should be disregarded for interpretation. Sample EKV26 failed C/N ratio control checks after AMS analysis, but passed after EA-IRMS analysis, so ^14^C data should thus be disregarded. The standard deviations of the samples’ ^14^C dates are generally low (about 15–35 years).

## Modelling: Stable isotopes

Ecological behaviours, such as diet and feeding depths, can strongly influence MREs. These can be studied through stable isotope analysis of the samples.

The exact species of geese could not be determined, but snow geese (Anser caerulescens), white fronted geese (Anser albifrons) and bean geese (Anser serrirostris) can be found on the Chukchi Peninsula. Based on present-day relative frequency, the samples are most likely snow geese (Portenko, 1981). The diets of the local goose species are all quite similar, feeding mainly on plant material. The goose samples are enriched in ^15^N (δ^15^N = +6.4‰ ± 0.9‰) relative to the other terrestrial species like hare (δ^15^N = +2.5‰ ± 1.0‰), reindeer (δ^15^N = +3.3‰ ± 1.6‰) or ptarmigan (δ^15^N = +1.6‰).

Hares have a similar diet to terrestrial-feeding geese. Their δ^13^C values are similar, though their δ^15^N values, are consistently lower than for geese. This could be due to their different physiology (e.g. their coprophagous feeding behaviour), the migratory behaviour of geese or the consumption of nitrogen-fixing plants. Reindeer mainly eat lichens in winter, explaining their higher δ^13^C values relative to geese and hare. Lichens are typically enriched in ^13^C and depleted in ^15^N relative to grasses (Lee et al., 2009). The isotopic measurements of the willow ptarmigan are consistent with other published ptarmigan data (Dehnhard et al., 2016), with a diet based mostly on berries, buds and leaves.

Stable isotopic data from the sampled marine species reflect a range of ecological behaviours. Walruses have lower δ^15^N values compared to the other marine mammals. They forage primarily in shallow coastal waters, consuming molluscs, but also shrimp, crabs, tube worms, soft corals, tunicates and sea cucumbers. Their reliance on shellfish explains their δ^15^N (δ^15^N = +13.1‰ ± 1.9‰); it has been demonstrated in other bodies of water that shellfish δ^15^N values do tend to be lower than other marine fauna (Richards and Hedges, 1999). These data are consistent with other published data from the Bering Strait and Chukchi Sea (Gorlova, 2014).

Ringed seal is a species adapted for sustained dives (Simpkins et al., 2001). Their dietary focus is fish, in particular cod, but also herring, smelt, whitefish, sculpin and perch (Chester, 2016) (occasionally they will also consume shrimp and crustaceans). Their stable isotopic composition is typical for piscivores, with higher δ^15^N values (+18.1‰ ± 1.7‰) compared to species such as walrus. The δ^13^C values (δ^13^C = −13.4‰ ± 1.0‰) are consistent with other published samples from the Bering Strait (Gorlova et al., 2012).

Bearded seals primarily feed on the sea bed, foraging for food in waters deeper than ringed seals or walrus. Of the marine mammals sampled, bearded seals feed in the deepest waters. Their search for food is aided by their ‘whiskers’ as they feel through the seafloor sediment. Bearded seals have been found to feed on invertebrates (such as anemones, sea cucumbers and polychaete worms) as well as sculpins and arctic cod (Finley and Evans, 1983). Whilst ringed seals focus on fish, and walruses focus on seafloor foraging, bearded seals consume a broad range of dietary components, consuming both pelagic and benthic species (Finley and Evans, 1983). This results in δ^15^N values (+16.2‰ ± 0.3‰, *N* = 2) intermediate to those of ringed seal (+18.1‰ ± 1.7‰, *N* = 6) and walrus (+13.1‰ ± 1.9‰, *N* = 3).

### Modelling: ^14^C dates and Δ*R* calculations

There is evidence for curation and long use-life of some classes of tools in the Siberian archaeological record (Pitulko, 2000), leading to the possibility of material predating the burial event being included in the grave context. Similarly, the effects of bioturbation and the inclusion of more recent material, need to be accounted for. In addition to our sampling strategy, which excluded artefacts or worked faunal elements, measures were taken to ensure that material with outlying dates was excluded.

Due to sampling constraints, for most grave contexts there were too few samples to perform χ^2^ tests. Instead, an OxCal model was developed to identify outlying samples (or those with a more complex depositional history) in the data set according to the following points:

- Each grave context was assigned a phase; in total 17 grave context phases were modelled. As the order of the burials was not known, they were modelled as ‘overlapping phases’ in that their start and end dates were independent of the other phases. Where both marine and terrestrial samples were present in a grave context, two further overlapping sub-phases were modelled within the ‘grave phase’.
- The marine reservoir effect was incorporated into the model by applying Δ*R* values to marine samples. Because the Δ*R* values were unknown, however, a linear Δ*R* range of between 0 and 800 years ‘U(0,800)’ was applied to all marine samples. From the published literature, it is clear that reservoir effects measured in marine species in the Bering Strait are in excess of the global average. The 800 years Δ*R* upper limit allowed for flexibility and the possibility of large MREs.
- Model fit was evaluated by examining the agreement indexes. The sample with the lowest agreement (below 60% agreement) was removed from the model and the model rerun until all remaining samples were in excess of 60% agreement (Bronk Ramsey, 2009b).

In total eight samples were found not to fit the parameters of the model and were removed from further analysis; three terrestrial samples (hare EKV16 and geese, EKV17 and EKV156), and five marine samples (walrus EKV01, gull EKV159, guillemot EKV18, ringed seal EKV20 and bearded seal EKV09). The model codes are presented in the supplementary information.

The methods presented here are designed to provide MRE estimates which are specific to different marine species. Paired terrestrial/marine sample analysis is a method used to calculate marine reservoir effect values (Ascough et al., 2005). This method is based on the assumption that organic material from a closed context will share the same calendar age. After eliminating the sampling of erroneously old material or recent material, and removing outlying dates, it is safely assumed that all terrestrial and marine material from a given grave context shares the same calendar age. Depending on the grave context, more than one terrestrial or marine sample was dated from a single feature. Where multiple terrestrial samples (of any species) were available for dating from a single grave context, an average ^14^C date was calculated according to methods detailed by Long and Rippeteau (1974).

To calculate a Δ*R* for each marine sample, the individual marine samples were compared to the grave’s terrestrial ^14^C date; all Δ*R* values were calculated using the Deltar tool (Reimer and Reimer, 2017), using the Marine20 curve (Heaton et al., 2020); the calibrated age of the terrestrial sample(s) is taken to be the best estimate of the grave context and, by association, the marine sample(s). As well as Δ*R* values, reservoir ages were calculated according to Soulet (2015).

## Discussion

### Δ*R* variability

Table 2 displays the range of Δ*R* values and reservoir ages from marine samples across the different grave contexts. The majority of samples have high MREs; samples that fit the OxCal model’s parameters range between Δ*R* = 136 ± 41 (reservoir age = 692 ± 28) and Δ*R* = 460 ± 40 (reservoir age = 971 ± 28). Although most MRE estimates appear quite similar, some need additional discussion.

**Table 2.** Sample ^14^C dates, Δ*R* values (using the Marine20 calibration curve) and reservoir ages.

Latin name	Common name	Burial no	Sample id	^14^C date (BP)	Δ*R* ± 1σ (Marine20)	*R*(*t*) reservoir age (years)
*Anser* sp.	Goose	214	EKV08	1295 ± 20		
*Cepphus columba*	Pigeon guillemot	214	EKV18	1875 ± 20	34 ± 43	580 ± 28
Lepus timidus	Hare	234	EKV19	1258 ± 19		
Pusa hispida	Ringed seal	234	EKV15	2155 ± 35	335 ± 49	897 ± 40
Lepus timidus	Mountain hare	249	EKV04	1170 ± 30		
*Erignathus barbatus*	Bearded seal	249	EKV09	2850 ± 20	1133 ± 56	1680 ± 46
*Lepus timidus*	Mountain hare	250	EKV16	3925 ± 25		
Rangifer tarandus	Reindeer	250	EKV22	1219 ± 19		
Pusa hispida	Ringed seal	250	EKV12	2140 ± 25	409 ± 43	921 ± 31
Pusa hispida	Ringed seal	250	EKV13	2140 ± 20	410 ± 40	921 ± 44
Odobenus rosmarus	Walrus	250	EKV14	2050 ± 25	319 ± 43	831 ± 31
Erignathus barbatus	Bearded seal	250	EKV11	2190 ± 20	460 ± 40	971 ± 28
Lepus timidus	Mountain hare	260	EKV39	1255 ± 25		
*Pusa hispida*	Ringed seal	260	EKV20	3355 ± 25	1537 ± 50	2100 ± 35
*Anser* sp.	Goose	279	EKV35	1345 ± 35		
*Odobenus rosmarus*	Walrus	279	EKV01	1580 ± 19	−307 ± 39	235 ± 40
*Anser* sp.	Goose	301	EKV33	1220 ± 20		
*Uria* sp.	Murre	301	EKV155	2024 ± 24	292 ± 45	804 ± 31
Lagopus lagopus	Willow ptarmigan	302	EKV37	1175 ± 30		
*Phalacrocorax* sp.	Cormorant	302	EKV157	1995 ± 24	278 ± 55	820 ± 38
*Larus* sp.	Gull	302	EKV159	1813 ± 13	91 ± 54	638 ± 33
*Anser* sp.	Goose	313	EKV10	1259 ± 19		
*Anser* sp.	Goose	313	EKV38	1236 ± 19		
	Average Terrestrial			1248 ± 13		
*Rissa* sp.	Kittiwake	313	EKV160	2070 ± 24	255 ± 41	822 ± 28
Lepus timidus	Mountain hare	317	EKV02	1186 ± 15		
Rangifer tarandus	Reindeer	317	EKV31	1241 ± 19		
	Average Terrestrial	317		1214 ± 12		
Pusa hispida	Ringed seal	317	EKV27	2025 ± 20	296 ± 34	811 ± 23
Pusa hispida	Ringed seal	317	EKV28	2010 ± 25	281 ± 37	796 ± 28
Odobenus rosmarus	Walrus	317	EKV29	1985 ± 20	256 ± 34	771 ± 23
*Anser* sp.	Goose	318	EKV25	1335 ± 20		
*Anser* sp.	Goose	318	EKV32	1300 ± 20		
	Average Terrestrial	318		1318 ± 14		
*Uria* sp.	Murre	318	EKV158	2010 ± 24	136 ± 41	692 ± 28
*Anser* sp.	Goose	222	EKV23	1315 ± 20		
*Anser* sp.	Goose	222	EKV24	1290 ± 20		
*Anser* sp.	Goose	293	EKV17	1065 ± 20		
*Anser* sp.	Goose	293	EKV156	1013 ± 24		
Lepus timidus	Mountain hare	310	EKV05	1225 ± 20		
Lepus timidus	Mountain hare	310	EKV06	1252 ± 19		
Lepus timidus	Mountain hare	310	EKV07	1290 ± 20		
*Anser* sp.	Goose	325	EKV34	1205 ± 20		
Rangifer tarandus	Reindeer	282	EKV03	1394 ± 19		
*Anser* sp.	Goose	283B	EKV36	1215 ± 25		

Samples identified as outliers are struck through.

The outlier model has most likely been successful in identifying faunal material not contemporaneous with the grave burial event. Polar and subpolar waters exhibit reservoir ages of up to 800–1200 years (Ascough et al., 2005). The reservoir ages for ringed seal EKV20 (2100 ± 35) in grave 260 and the bearded seal EKV09 (1680 ± 46) in grave 249 (Δ*R* = 1537 ± 50 and 1133 ± 56 respectively) are extremely high and were identified as outliers. These values are most likely due to old faunal material being incorporated into the grave context during the burial process or disturbed by later mortuary activity. The Δ*R* value of the walrus sample EKV01 differs in that it is the only Δ*R* value (Δ*R* = −307 ± 39) which is lower than the global average, its *R*(*t*) is similarly low (=235 ± 40). This sample has been identified as an outlier by the OxCal model. To ensure that it was the walrus sample, and not the goose, in grave 279 that was the cause of this unusual Δ*R* value, a second OxCal model was constructed (Appendix 1). This model calibrated all terrestrial samples in a single phase with an outlier test; the goose sample (EKV35) was not identified as an outlier among the other terrestrial samples, demonstrating its fit within the terrestrial dataset. Whilst it is possible for a reservoir age of 235 ± 40 years (Δ*R* = −307 ± 39) to be measured in a marine sample, given the poor statistical fit of the ^14^C date of EKV01 among the other fauna, it is more likely the sample became incorporated into the grave context through bioturbation or animal action.

It is perhaps not surprising that these samples (EKV01, EKV09 and EKV20) were identified as outliers. When compared with ‘contemporaneous’ terrestrial material, their Δ*R* values fell outside the Δ*R* = 0–800 prior added to marine samples in the OxCal model. Although these marine and terrestrial samples were recovered from the same grave contexts as other faunal material, it is unlikely these animals were contemporaneous. Rather, it is more likely that these three calculated Δ*R* values are not representative of different radiocarbon values in terrestrial and marine environments, but the result of complex deposition histories of these samples. The Δ*R* range of 0–800 was selected to be both wide, allowing for flexible MRE possibilities whilst ^14^C dates were calibrated, and also to be consistent with expected MRE observations from the Bering Strait.

**Figure 2.**
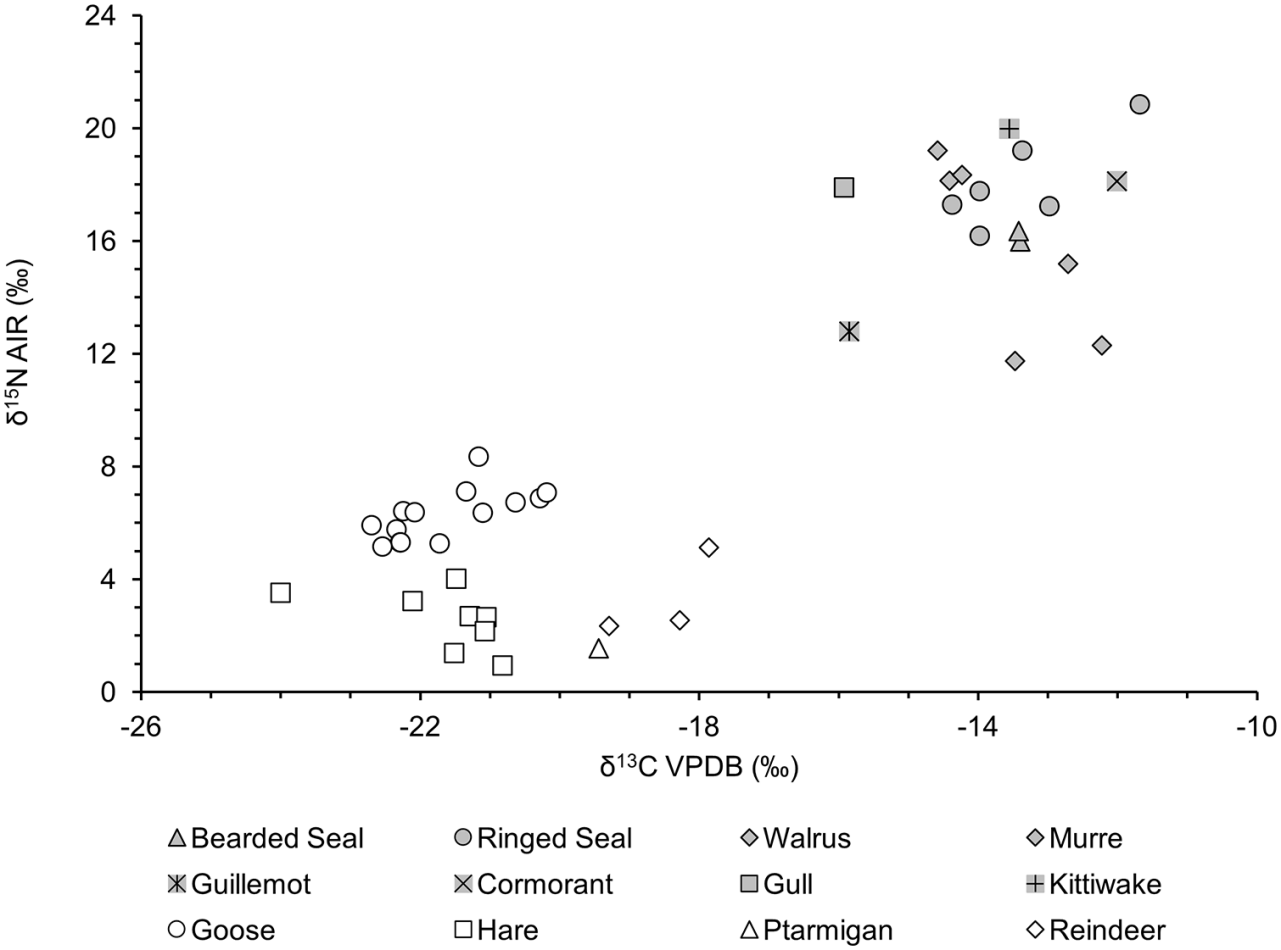
δ^15^N and δ^13^C values for samples of marine and terrestrial fauna from Ekven.

Also identified as an outlier, the guillemot sample (EKV18) has a Δ*R* value much lower than the other samples (Δ*R* = 34 ± 43). Although this is a marine bird and has been treated as marine fauna within the model, a small amount of its feeding activity does take place on land. Guillemots are known to consume moderate amounts of insects and plant material in addition to their dietary staple of fish and molluscs. As previously discussed, looking at the stable isotopic data of EKV18 (Figure 2), this is evident. Although the δ^15^N of this sample is comparable to those of the walrus samples, its δ^13^C is lower than the rest of the marine samples. The stable isotopic data for this sample point towards it being a marine feeder with occasional (yet isotopically significant) dietary inputs from terrestrial sources. Its mixed terrestrial/marine diet, however, would lead this sample to show <100% of the local marine reservoir effect.

If the marine samples which do not fit the model parameters are disregarded, the average Δ*R* value of the marine faunal material recovered from Ekven is 306 ± 87 years (an average reservoir age of 825 ± 77 years). The OxCal model, it should be understood, does not identify erroneous ^14^C dates within a dataset, rather it highlights outliers in a data set that need further consideration.

These values have far-reaching implications for the archaeology of Ekven and beyond. It has been demonstrated that in the Bering Sea, and other locations (Russell et al., 2011a), different species living in the same water can yield different reservoir ages and Δ*R* values. For those wishing to understand the full range of MREs within a given location, these data make it clear that analysis of a single species will not suffice. In addition to the MRE measurements calculated as part of this study, 84 additional relevant samples from a greater number of marine species have been included in Table 3. MRE measurements, based on published ^14^C dates, have been calculated using a sample pairing method or known collection date. The samples are from coastal sites along the Chukchi and Bering Seas from the last 2000 years (temporal variability across this dataset will be investigated).

**Table 3.** Δ*R* values and reservoir ages of marine samples from coastal Chukotka and Alaska.

Sample location	Marine sample (common name)	^14^C date (marine sample)	Contemporary terrestrial sample(s)	^14^C date terrestrial sample(s)	Terrestrial sample calibrated/collection date AD	Δ*R* ± σ (Marine20)	*R*(*t*) reservoir age (years)	Publication
Nash Harbor	Common mussel	775 ± 50	Charcoal	205 ± 40	1637–1950	−14 ± 84*	570 ± 64	Dumond and Griffin (2002)
Nash Harbor	Common mussel	725 ± 50	Charcoal	335 ± 40	1466–1643	−221 ± 75*	390 ± 64	Dumond and Griffin (2002)
Nash Harbor	Common mussel	805 ± 50	Charcoal	245 ± 40	1514–1950	−49 ± 82*	560 ± 64	Dumond and Griffin (2002)
Nash Harbor	Common mussel	830 ± 50	Charcoal	950 ± 40	1021–1205	−647 ± 68*	−120 ± 64	Dumond and Griffin (2002)
Nash Harbor	Common mussel	840 ± 50	Charcoal	505 ± 45	1320–1463	−272 ± 64*	335 ± 67	Dumond and Griffin (2002)
Port Clarence	Common mussel	800 ± 50			1913	193 ± 51	680 ± 50	McNeely et al. (2006)
Port Clarence	Common mussel	975 ± 20			1913	368 ± 20	855 ± 20	McNeely et al. (2006)
Point Barrow	Common mussel	870 ± 40			1913	263 ± 41	750 ± 40	McNeely et al. (2006)
Teller	Arctic hiatella	1030 ± 40			1913	423 ± 41	910 ± 40	McNeely et al. (2006)
Teller	Arctic hiatella	900 ± 20			1913	293 ± 20	780 ± 20	McNeely et al. (2006)
Pavlov Harbor	Littleneck clam	700 ± 50			1937	93 ± 51	539 ± 51	Robinson and Thompson (1981)
Nash Harbor	Walrus	965 ± 50	Charcoal	205 ± 40	1637–1950	176 ± 84*	760 ± 64	Dumond and Griffin (2002)
Nash Harbor	Walrus	1170 ± 50	Charcoal	335 ± 40	1466–1643	224 ± 75*	835 ± 64	Dumond and Griffin (2002)
Nash Harbor	Walrus	1075 ± 50	Charcoal	370 ± 40	1449–1635	92 ± 78*	705 ± 64	Dumond and Griffin (2002)
Nash Harbor	Walrus	1200 ± 50	Charcoal	445 ± 45	1404–1623	137 ± 66*	755 ± 67	Dumond and Griffin (2002)
Ekven	Walrus	2050 ± 25	Reindeer	1219 ± 19	707–883	319 ± 43	831 ± 31	This Study
Ekven	Walrus	1985 ± 20	Mountain hare/Reindeer*	1214 ± 12	785–878	256 ± 34	771 ± 23	This Study
Walakpa	Ringed seal	1810 ± 29	Caribou*	1003 ± 20	991–1148	264 ± 39	807 ± 35	Krus et al. (2019)
Walakpa	RInged seal	1761 ± 27	Caribou*	1003 ± 20	991–1148	215 ± 37	758 ± 34	Krus et al. (2019)
Walakpa	Ringed seal	1637 ± 29	Caribou*	714 ± 18	1270–1300	356 ± 32	923 ± 34	Krus et al. (2019)
Walakpa	Ringed seal	1694 ± 33	Caribou*	714 ± 18	1270–1300	412 ± 35	980 ± 38	Krus et al. (2019)
Cape Espenberg	Ringed Seal	1671 ± 45	Caribou	683 ± 7	1281–1378	403 ± 47	988 ± 46	Reuther et al. (2021)
Deering	Ringed Seal	2007 ± 46	Caribou/Charcoal	1256 ± 23	673–866	193 ± 63	751 ± 52	Reuther et al. (2021)
Deering	Ringed Seal	1680 ± 28	Caribou/Charcoal	873 ± 17	1159–1220	291 ± 35	807 ± 33	Reuther et al. (2021)
Deering	Ringed Seal	1718 ± 51	Caribou/Wood	811 ± 25	1180–1275	380 ± 55	907 ± 57	Reuther et al. (2021)
Deering	Ringed Seal	1682 ± 45	Caribou/Wood	811 ± 25	1180–1275	344 ± 50	871 ± 51	Reuther et al. (2021)
Kotzebue	Ringed Seal	1537 ± 48	Caribou	718 ± 26	1262–1381	254 ± 52	819 ± 55	Reuther et al. (2021)
Deering	Ringed Seal	1633 ± 32	Caribou/Charcoal	873 ± 17	1159–1220	244 ± 39	760 ± 36	Reuther et al. (2021)
Deering	Ringed Seal	1669 ± 40	Caribou/Charcoal	873 ± 17	1159–1220	279 ± 46	796 ± 43	Reuther et al. (2021)
Ekven	Ringed seal	2155 ± 35	Mountain hare	1258 ± 19	676–824	335 ± 49	897 ± 40	This Study
Ekven	Ringed seal	2140 ± 25	Reindeer	1219 ± 19	707–883	409 ± 43	921 ± 31	This Study
Ekven	Ringed seal	2140 ± 20	Reindeer	1219 ± 19	707–883	410 ± 40	921 ± 28	This Study
Ekven	Ringed seal	2025 ± 20	Mountain hare/Reindeer*	1214 ± 12	785–878	296 ± 34	811 ± 23	This Study
Ekven	Ringed seal	2010 ± 25	Mountain hare/Reindeer*	1214 ± 12	785–878	281 ± 37	796 ± 28	This Study
Ekven	Bearded seal	2190 ± 20	Reindeer	1219 ± 19	707–883	460 ± 40	971 ± 28	This Study
Cape Krusenstern	Bearded seal	1550 ± 30	Reindeer	840 ± 24	1168–1263	186 ± 42	710 ± 39	Reuther et al. (2021)
Cape Krusenstern	Bearded seal	2230 ± 30	Charcoal	1590 ± 39	413–564	155 ± 50	640 ± 50	Reuther et al. (2021)
Kivalina	Bearded seal	2262 ± 47	Reindeer	1470 ± 40	545–652	293 ± 57	792 ± 62	Reuther et al. (2021)
Kotzebue	Bearded seal	1150 ± 20	Reindeer	340 ± 20	1479–1635	210 ± 60	920 ± 28	Reuther et al. (2021)
Kivalina	Spotted Seal/Harbour Seal	2340 ± 47	Reindeer	1470 ± 40	545–652	371 ± 57	870 ± 62	Reuther et al. (2021)
Kivalina	Spotted Seal/Harbour Seal	2327 ± 47	Reindeer	1470 ± 40	545–652	358 ± 57	857 ± 62	Reuther et al. (2021)
Kivalina	Spotted Seal/Harbour Seal	2336 ± 47	Reindeer	1470 ± 40	545–652	367 ± 57	866 ± 62	Reuther et al. (2021)
Kotzebue	Spotted Seal/Harbour Seal	1642 ± 48	Reindeer	718 ± 26	1262–1381	359 ± 52	924 ± 55	Reuther et al. (2021)
Nash Harbor	Unknown phocid	1230 ± 50	Charcoal	460 ± 50	1328–1623	155 ± 68*	770 ± 71	Dumond and Griffin (2002)
Nash Harbor	Unknown phocid	1165 ± 50	Charcoal	950 ± 40	1021–1205	−312 ± 68*	215 ± 64	Dumond and Griffin (2002)
Cape Prince of Wales	Unknown phocid	1100 ± 50	Peat	460 ± 50	1328–1623	25 ± 68*	640 ± 71	Dumond and Griffin (2002)
Cape Prince of Wales	Unknown phocid	1220 ± 40	Peat/Grass*	590 ± 42	1299–1421	33 ± 58*	630 ± 58	Dumond and Griffin (2002)
Cape Espenberg	Unknown Seal	1343 ± 28	Caribou/charcoal	383 ± 12	1455–1618	322 ± 37	960 ± 30	Reuther et al. (2021)
Cape Espenberg	Unknown Seal	1422 ± 30	Caribou/charcoal	497 ± 13	1409–1442	321 ± 34	925 ± 36	Reuther et al. (2021)
Cape Espenberg	Unknown Seal	1599 ± 45	Caribou	683 ± 7	1281–1378	311 ± 47	916 ± 46	Reuther et al. (2021)
Cape Krusenstern	Unknown Seal	880 ± 30	Caribou	60 ± 30	1694–1918		820 ± 42	Reuther et al. (2021)
Cape Krusenstern	Unknown Seal	810 ± 30	Caribou	210 ± 30	1644–1950	22 ± 80	600 ± 42	Reuther et al. (2021)
Cape Krusenstern	Unknown Seal	1020 ± 30	Charcoal	400 ± 40	1434–1632	−6 ± 58	620 ± 50	Reuther et al. (2021)
Cape Krusenstern	Unknown Seal	1110 ± 30	Charcoal	570 ± 40	1302–1428	−63 ± 53	540 ± 50	Reuther et al. (2021)
Cape Krusenstern	Unknown Seal	1170 ± 30	Charcoal	280 ± 40	1486–1798	269 ± 63	890 ± 50	Reuther et al. (2021)
Cape Krusenstern	Unknown Seal	1920 ± 30	Charcoal	1200 ± 40	686–971	191 ± 62	720 ± 50	Reuther et al. (2021)
Cape Krusenstern	Unknown Seal	1450 ± 30	Caribou	510 ± 29	1329–1449	340 ± 38	940 ± 42	Reuther et al. (2021)
Cape Krusenstern	Unknown Seal	1410 ± 30	Caribou	640 ± 29	1286–1397	187 ± 50	770 ± 42	Reuther et al. (2021)
Cape Krusenstern	Unknown Seal	1280 ± 30	Charcoal	765 ± 34	1220–1287	−37 ± 39	515 ± 46	Reuther et al. (2021)
Deering	Unknown Seal	2024 ± 46	Caribou/Charcoal	1256 ± 24	673–866	210 ± 63	768 ± 52	Reuther et al. (2021)
Kotzebue	Unknown Seal	1150 ± 20	Caribou	230 ± 20	1640–1800	315 ± 30	872 ± 28	Reuther et al. (2021)
Maiyumerak Creek	Unknown Seal	1350 ± 20	Caribou	274 ± 21	1522–1794	461 ± 62	1076 ± 29	Reuther et al. (2021)
Naknek River	Beluga whale	1040 ± 74	Charcoal	240 ± 50	1497–1950	193 ± 110*	800 ± 89	Dumond and Griffin (2002)
Summer Bay	Unknown whale	2480 ± 70	Charcoal*	1975 ± 42	51 BC–201	35 ± 84*	505 ± 82	Dumond and Griffin (2002)
Gambell	Unknown whale	1948 ± 78	Wood	1270 ± 86	611–975	147 ± 117*	678 ± 116	Dumond and Griffin (2002)
Gambell	Unknown whale	1908 ± 78	Wood	1530 ± 94	262–664	−128 ± 113*	378 ± 122	Dumond and Griffin (2002)
Gambell	Unknown whale	1588 ± 108	Wood	1100 ± 86	691–1156	−54 ± 144*	488 ± 87	Dumond and Griffin (2002)
Gambell	Unknown whale	1528 ± 84	Wood	940 ± 78	979–1265	59 ± 112*	588 ± 115	Dumond and Griffin (2002)
Gambell	Unknown whale	1298 ± 84	Wood	990 ± 86	882–1252	−214 ± 123*	308 ± 120	Dumond and Griffin (2002)
Gambell	Unknown whale	1288 ± 92	Wood	460 ± 86	1308–1639	221 ± 130*	828 ± 126	Dumond and Griffin (2002)
Ekven	Murre	2024 ± 24	Goose	1220 ± 20	706–883	293 ± 45	804 ± 31	This Study
Ekven	Murre	2010 ± 24	Goose	1318 ± 14	658–774	136 ± 41	692 ± 28	This Study
Ekven	Cormorant	1995 ± 24	Ptarmigan	1175 ± 30	772–973	278 ± 55	820 ± 38	This Study
Ekven	Kittiwake	2070 ± 24	Goose*	1248 ± 13	682–828	255 ± 41	822 ± 27	This Study

Source: Δ*R* values re-calculated from published sources using the Deltar tool (Reimer and Reimer, 2017) against the Marine20 curve (Heaton et al., 2020). Reservoir ages calculated according to Soulet (2015). Samples marked with (*) are averages of multiple ^14^C dates (Long and Rippeteau, 1974).

**Figure 3.**
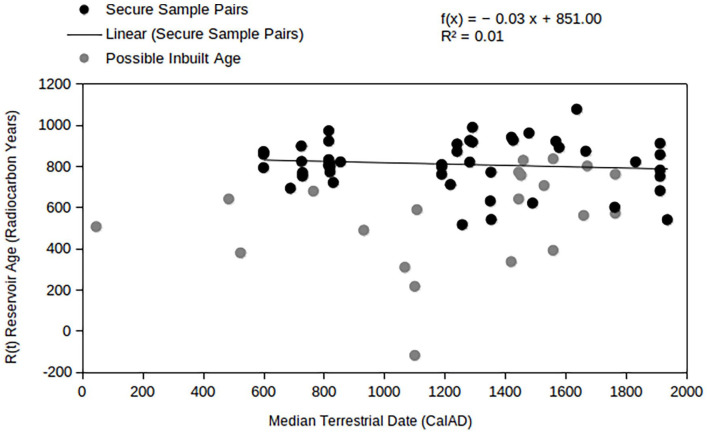
Marine sample reservoir age values against the median calibrated date of the corresponding terrestrial samples (Table 3).

These data highlight further the importance of selecting an appropriate terrestrial sample for the determination of the MRE. Figure 3 displays the samples’ marine reservoir age values against their associated terrestrial ^14^C dates (or collection year). The data set is organised into two groups, those which are likely to be subject to the old-wood problem, that is, charcoal and wood, and those calculated with a secure terrestrial material, that is, bone or charred twigs (note that some papers account for the old-wood problem through chi-squared tests, these sample pairs are considered ‘secure’). The charcoal/wood samples show significantly smaller reservoir ages. Though not all wood/charcoal and marine pairings will lead to erroneously low MRE estimates, this data illustrates that it is likely in this context. Although MREs can change through time, it appears that the MREs measured in marine samples from the wider region have remained constant. Considering only the ‘secure’ pairings, a linear regression line shows only a very slight increase in the reservoir age of marine samples over time (with a low *R*^2^ value of 0.01, the time period is likely not an important variable in this context).

**Figure 4.**
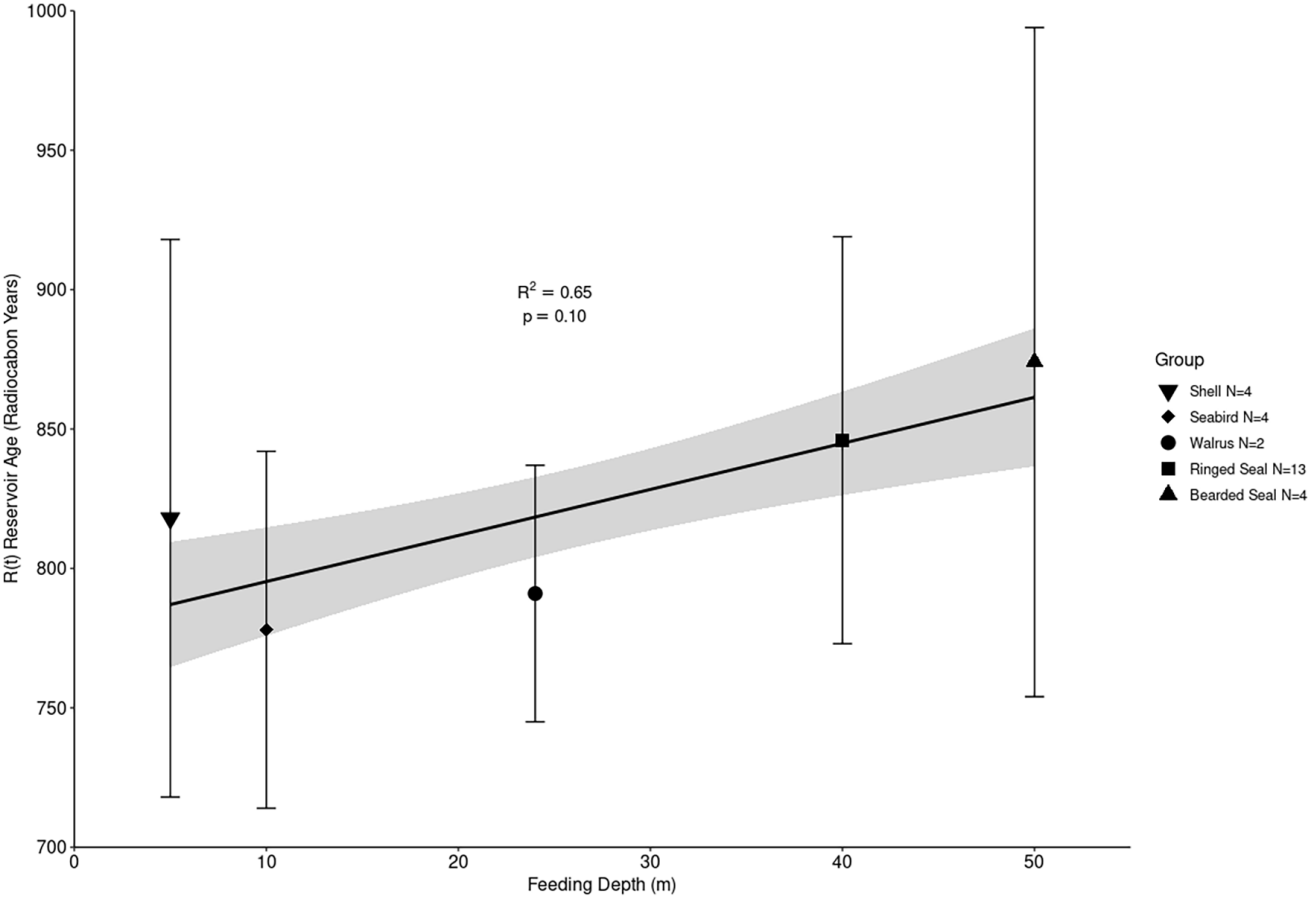
Reservoir age against estimated feeding depth of marine fauna. The shaded area around the linear regression line represents a ∙1 σ probability range.

There is an inverse relationship between the abundance of ^14^C and the distance from the surface (Dumond and Griffin, 2002). Marine samples from surface waters yield reservoir ages of roughly 400 years, whereas samples from deeper waters can have much larger MREs (Oeschger et al., 1975; Stuiver and Braziunas, 1993). To explore one variable in MRE, Figure 4 displays the average reservoir age of a species/taxa against an estimate of the feeding depth. Here, feeding depths are limited to 50 m, the maximum depth of the Bering Strait. All samples identified as outliers, samples of unknown species and ‘unsecure’ paired wood/charcoal samples are excluded. Of the marine mammals studied, bearded seals feed at the greatest depths. They feed on the seafloor at depths of roughly 100 m (Kovacs, 2018). Though ringed seals can feed in deep waters, they typically feed at shallower depths than bearded seals, hunting fish rather than seafloor bivalves (Chester, 2016). Walrus can dive at depths up to 100 m (Fay and Burns, 1988) but tend to feed in much shallower shoreline waters, 24 m on average (Schreer et al., 2001). All the shellfish samples are known to have been collected from shallow waters on the immediate coastline (here a depth of 5 m has been applied), however, the species listed can occupy deeper waters. Three species of seabirds were sampled in this study. Murre and cormorants are adept divers, however, the majority of their target prey occupy surface waters at shallow depths. Kittiwakes do not dive in the same fashion and collect their prey from the very surface. Considering this variation, a feeding depth of 10 m was applied.

The *R*(t) values calculated for the various marine species along the Chukchi and Alaskan coasts appear to be positively correlated with feeding depth, although the relationship is not statistically significant (*R*^2^ = 0.65, *p* = 0.10). Clearly, feeding depth alone cannot account for the complex MRE variability among the sampled species. These data demonstrate that feeding depth is likely one factor in a species average measured reservoir effect; other ecological variables such as migration most likely also contribute.

### Archaeological implications

In addition to detailing MRE variability among marine species, these calculations also serve to calculate an appropriate Δ*R* value to apply to ^14^C dates of human skeletal remains from the Ekven mortuary site. The OBS inhabitants of the area around Ekven and the Chukchi coast were almost entirely reliant on marine resources. The Δ*R* and *R*(t) values, listed in Table 2, demonstrate the range of marine reservoir effects across several different species available to those living near Ekven. Depending on the diets and hunting practices of OBS peoples at Ekven, these data demonstrate the importance of selecting appropriate MRE corrections for the calibration of ^14^C ages from human bone collagen. Evidence from zooarchaeological assemblages and material culture recovered from many OBS sites suggest the importance of seal and walrus to human diets in this region. Using Δ*R* values of all seal species (including walrus) from Table 3, an average marine mammal Δ*R* value of 289 ± 124 years and an *R*(t) of 842 ± 123, is calculated. These would be most appropriate MRE corrections to apply to calibration of ^14^C dated human bones from Ekven and the Bering Strait region. MRE correction values may be weighted as more data (including human bone collagen stable isotopic data) becomes available. These may indicate if one species of marine mammal was predominant.

## Conclusions

The site of Ekven provides an opportunity to demonstrate the variability of reservoir effects between species in a given geographical area. Using paired dating of terrestrial and marine faunal samples from secure grave contexts, radiocarbon dates were used to estimate reservoir effects across seven different marine species. These measurements, in addition to data from other published sources, demonstrate a range of MREs across marine species. Although it had already been demonstrated that the MRE was quite substantial around the wider Bering Strait region (Dumond and Griffin, 2002; Krus et al., 2019; West et al., 2019), it remained unclear how the marine ^14^C reservoir affected various species. Those conducting radiocarbon dating of human remains from OBS sites like Ekven (and other sites around the Bering Strait) should consider the variability of Δ*R* values before selecting the most appropriate. Archaeological evidence indicates seal and walrus were the main dietary resource of those buried at Ekven. We, therefore, suggest a Δ*R* (Marine20) value of 289 ± 124 years, or reservoir age of 842 ± 123, for this key site. The MRE corrections applied to other cultural groups around the Bering Strait will of course differ, depending on diet. Beyond Ekven and the Bering Strait, we encourage the calculation of local MRE corrections which are both species-specific and consider culture-specific dietary regimes.

## Supplemental Material

sj-docx-1-hol-10.1177_09596836211041728 – Supplemental material for Species-specific reservoir effect estimates: A case study of archaeological marine samples from the Bering Strait Click here for additional data file. Supplemental material, sj-docx-1-hol-10.1177_09596836211041728 for Species-specific reservoir effect estimates: A case study of archaeological marine samples from the Bering Strait by Jack PR Dury, Gunilla Eriksson, Arkady Savinetsky, Maria Dobrovolskaya, Kirill Dneprovsky, Alison JT Harris, Johannes van der Plicht, Peter Jordan and Kerstin Lidén in The Holocene
