# Supplementary material for: Species-specific reservoir effect estimates: A case study of archaeological marine samples from the Bering Strait
Source: Holocene. 2021 Sep 6;32(11):1209–21. doi: 10.1177/09596836211041728 (PMC9511246; doi:10.1177/09596836211041728)
Supplement: sj-docx-1-hol-10.1177_09596836211041728 – Supplemental material for Species-specific reservoir effect estimates: A case study of archaeological marine samples from the Bering Strait [file sj-docx-1-hol-10.1177_09596836211041728.docx]

**Appendix- OxCal Model Code**

Plot(Marine and Terrestrial Outlier Model)

{

Options()

{

Resolution=1;

};

Outlier_Model("General",T(5),U(0,4),"t");

Phase()

{

Sequence()

{

Boundary("Start 1");

Phase("1")

{

Phase()

{

Sequence()

{

Boundary("Start 1");

Phase("1")

{

Phase()

{

Sequence()

{

Boundary("Start 1");

Phase("1")

{

Curve("Marine20","Marine20.14c"); Delta_R("LocalMarine",U(0,800));

R_Date("EKV18", 1875, 20)

{

Outlier(0.05);

};

};

Boundary("End 1");

};

Sequence()

{

Boundary("Start 2");

Phase("2")

{

R_Date("EKV08", 1295, 20)

{

Outlier(0.05);

};

};

Boundary("End 2");

};

};

};

Boundary("End 1");

};

Sequence()

{

Boundary("Start 2");

Phase("2")

{

Phase()

{

Sequence()

{

Boundary("Start 1");

Phase("1")

{

Curve("Marine20","Marine20.14c");

Delta_R("LocalMarine",U(0,800));

R_Date("EKV15", 2155, 35)

{

Outlier(0.05);

};

};

Boundary("End 1");

};

Sequence()

{

Boundary("Start 2");

Phase("2")

{

R_Date("EKV19", 1258, 19)

{

Outlier(0.05);

};

};

Boundary("End 2");

};

};

};

Boundary("End 2");

};

Sequence()

{

Boundary("Start 3");

Phase("3")

{

Phase()

{

Sequence()

{

Boundary("Start 1");

Phase("1")

{

Curve("Marine20","Marine20.14c");

Delta_R("LocalMarine",U(0,800));

R_Date("EKV09", 2850, 20)

{

Outlier(0.05);

};

};

Boundary("End 1");

};

Sequence()

{

Boundary("Start 2");

Phase("2")

{

R_Date("EKV04", 1170, 30)

{

Outlier(0.05);

};

};

Boundary("End 2");

};

};

};

Boundary("End 3");

};

Sequence()

{

Boundary("Start 4");

Phase("4")

{

Phase()

{

Sequence()

{

Boundary("Start 1");

Phase("1")

{

Curve("Marine20","Marine20.14c");

Delta_R("LocalMarine",U(0,800));

R_Date("EKV12", 2140, 25)

{

Outlier(0.05);

};

R_Date("EKV13", 2140, 20)

{

Outlier(0.05);

};

R_Date("EKV14", 2050, 25)

{

Outlier(0.05);

};

R_Date("EKV11", 2190, 20)

{

Outlier(0.05);

};

};

Boundary("End 1");

};

Sequence()

{

Boundary("Start 2");

Phase("2")

{

R_Date("EKV16", 3925, 25)

{

Outlier(0.05);

};

R_Date("EKV22", 1219, 19)

{

Outlier(0.05);

};

};

Boundary("End 2");

};

};

};

Boundary("End 4");

};

Sequence()

{

Boundary("Start 5");

Phase("5")

{

Phase()

{

Sequence()

{

Boundary("Start 1");

Phase("1")

{

Curve("Marine20","Marine20.14c");

Delta_R("LocalMarine",U(0,800));

R_Date("EKV20", 3355, 25)

{

Outlier(0.05);

};

};

Boundary("End 1");

};

Sequence()

{

Boundary("Start 2");

Phase("2")

{

R_Date("EKV39", 1255, 25)

{

Outlier(0.05);

};

};

Boundary("End 2");

};

};

};

Boundary("End 5");

};

Sequence()

{

Boundary("Start 6");

Phase("6")

{

Phase()

{

Sequence()

{

Boundary("Start 1");

Phase("1")

{

Curve("Marine20","Marine20.14c");

Delta_R("LocalMarine",U(0,800));

R_Date("EKV01", 1580, 19)

{

Outlier(0.05);

};

};

Boundary("End 1");

};

Sequence()

{

Boundary("Start 2");

Phase("2")

{

R_Date("EKV35", 1345, 35)

{

Outlier(0.05);

};

};

Boundary("End 2");

};

};

};

Boundary("End 6");

};

Sequence()

{

Boundary("Start 7");

Phase("7")

{

Phase()

{

Sequence()

{

Boundary("Start 1");

Phase("1")

{

Curve("Marine20","Marine20.14c");

Delta_R("LocalMarine",U(0,800));

R_Date("EKV27", 2025, 20)

{

Outlier(0.05);

};

R_Date("EKV28", 2010, 25)

{

Outlier(0.05);

};

R_Date("EKV29", 1985, 20)

{

Outlier(0.05);

};

};

Boundary("End 1");

};

Sequence()

{

Boundary("Start 2");

Phase("2")

{

R_Date("EKV02", 1186, 15)

{

Outlier(0.05);

};

R_Date("EKV31", 1241, 19)

{

Outlier(0.05);

};

};

Boundary("End 2");

};

};

};

Boundary("End 7");

};

Sequence()

{

Boundary("Start 8");

Phase("8")

{

R_Date("EKV23G", 1315, 20)

{

Outlier(0.05);

};

R_Date("EKV24G", 1290, 20)

{

Outlier(0.05);

};

};

Boundary("End 8");

};

Sequence()

{

Boundary("Start 9");

Phase("9")

{

R_Date("EKV05G", 1225, 20)

{

Outlier(0.05);

};

R_Date("EKV06G", 1252, 19)

{

Outlier(0.05);

};

R_Date("EKV07", 1290, 20)

{

Outlier(0.05);

};

};

Boundary("End 9");

};

Sequence()

{

Boundary("Start 12");

Phase("12")

{

R_Date("EKV34", 1205, 20)

{

Outlier(0.05);

};

};

Boundary("End 12");

};

Sequence()

{

Boundary("Start 15");

Phase("15")

{

R_Date("EKV03", 1394, 19)

{

Outlier(0.05);

};

};

Boundary("End 15");

};

Sequence()

{

Boundary("Start 16");

Phase("16")

{

R_Date("EKV17", 1065, 20)

{

Outlier(0.05);

};

R_Date("EKV156", 1013, 24)

{

Outlier(0.05);

};

};

Boundary("End 16");

};

Sequence()

{

Boundary("Start 17");

Phase("17")

{

R_Date("EKV36", 1215, 25)

{

Outlier(0.05);

};

};

Boundary("End 17");

};

Sequence()

{

Boundary("Start 13");

Phase("13")

{

Phase()

{

Sequence()

{

Boundary("Start 1");

Phase("1")

{

Curve("Marine20","Marine20.14c");

Delta_R("LocalMarine",U(0,800));

R_Date("EKV155", 2024, 24)

{

Outlier(0.05);

};

};

Boundary("End 1");

};

Sequence()

{

Boundary("Start 2");

Phase("2")

{

R_Date("EKV33G", 1220, 20)

{

Outlier(0.05);

};

};

Boundary("End 2");

};

};

};

Boundary("End 13");

};

Sequence()

{

Boundary("Start 14");

Phase("14")

{

Phase()

{

Sequence()

{

Boundary("Start 1");

Phase("1")

{

Curve("Marine20","Marine20.14c");

Delta_R("LocalMarine",U(0,800));

R_Date("EKV157", 1995, 24)

{

Outlier(0.05);

};

R_Date("EKV159", 1813, 24)

{

Outlier(0.05);

};

};

Boundary("End 1");

};

Sequence()

{

Boundary("Start 2");

Phase("2")

{

R_Date("EKV37", 1175, 30)

{

Outlier(0.05);

};

};

Boundary("End 2");

};

};

};

Boundary("End 14");

};

Sequence()

{

Boundary("Start 10");

Phase("10")

{

Phase()

{

Sequence()

{

Boundary("Start 1");

Phase("1")

{

Curve("Marine20","Marine20.14c");

Delta_R("LocalMarine",U(0,800));

R_Date("EKV160", 2070, 24)

{

Outlier(0.05);

};

};

Boundary("End 1");

};

Sequence()

{

Boundary("Start 2");

Phase("2")

{

R_Date("EKV10", 1259, 19)

{

Outlier(0.05);

};

R_Date("EKV38", 1236, 19)

{

Outlier(0.05);

};

};

Boundary("End 2");

};

};

};

Boundary("End 10");

};

Sequence()

{

Boundary("Start 11");

Phase("11")

{

Phase()

{

Sequence()

{

Boundary("Start 1");

Phase("1")

{

Curve("Marine20","Marine20.14c");

Delta_R("LocalMarine",U(0,800));

R_Date("EKV158", 2010, 24)

{

Outlier(0.05);

};

};

Boundary("End 1");

};

Sequence()

{

Boundary("Start 2");

Phase("2")

{

R_Date("EKV25", 1334, 20)

{

Outlier(0.05);

};

R_Date("EKV32", 1300, 20)

{

Outlier(0.05);

};

};

Boundary("End 2");

};

};

};

Boundary("End 11");

};

};

};

Boundary("End 1");

};

};

};

Plot(Terrestrial Outlier Model)

{

Options()

{

Resolution=1;

};

Outlier_Model("General",T(5),U(0,4),"t");

Sequence()

{

Boundary("Start 1");

Phase("1")

{

R_Date("EKV08", 1295, 20)

{

Outlier("Outlier", 0.05);

};

R_Date("EKV23", 1315, 20)

{

Outlier("Outlier", 0.05);

};

R_Date("EKV24", 1290, 20)

{

Outlier("Outlier", 0.05);

};

R_Date("EKV35", 1345, 35)

{

Outlier("Outlier", 0.05);

};

R_Date("EKV17", 1065, 20)

{

Outlier("Outlier", 0.05);

};

R_Date("EKV33", 1220, 20)

{

Outlier("Outlier", 0.05);

};

R_Date("EKV10", 1259, 19)

{

Outlier("Outlier", 0.05);

};

R_Date("EKV38G", 1236, 19)

{

Outlier("Outlier", 0.05);

};

R_Date("EKV25", 1335, 20)

{

Outlier("Outlier", 0.05);

};

R_Date("EKV32", 1300, 20)

{

Outlier("Outlier", 0.05);

};

R_Date("EKV34", 1205, 20)

{

Outlier("Outlier", 0.05);

};

R_Date("EKV36", 1215, 25)

{

Outlier("Outlier", 0.05);

};

R_Date("EKV22", 1219, 19)

{

Outlier("Outlier", 0.05);

};

R_Date("EKV03", 1394, 19)

{

Outlier("Outlier", 0.05);

};

R_Date("EKV31", 1241, 19)

{

Outlier("Outlier", 0.05);

};

R_Date("EKV19", 1258, 19)

{

Outlier("Outlier", 0.05);

};

R_Date("EKV04", 1170, 30)

{

Outlier("Outlier", 0.05);

};

R_Date("EKV16", 3925, 25)

{

Outlier("Outlier", 0.05);

};

R_Date("EKV39", 1255, 25)

{

Outlier("Outlier", 0.05);

};

R_Date("EKV05", 1225, 20)

{

Outlier("Outlier", 0.05);

};

R_Date("EKV06", 1252, 19)

{

Outlier("Outlier", 0.05);

};

R_Date("EKV07", 1290, 20)

{

Outlier("Outlier", 0.05);

};

R_Date("EKV02", 1186, 15)

{

Outlier("Outlier", 0.05);

};

R_Date("EKV156", 1013, 24)

{

Outlier(0.05);

};

};

Boundary("End 1");

};

};
